# Colonization with the enteric protozoa *Blastocystis* is associated with increased diversity of human gut bacterial microbiota

**DOI:** 10.1038/srep25255

**Published:** 2016-05-05

**Authors:** Christophe Audebert, Gaël Even, Amandine Cian, The Blastocystis Investigation Group, Dima El Safadi, Gabriela Certad, Laurence Delhaes, Bruno Pereira, Céline Nourrisson, Philippe Poirier, Ivan Wawrzyniak, Frédéric Delbac, Christelle Morelle, Patrick Bastien, Laurence Lachaud, Anne-Pauline Bellanger, Françoise Botterel, Ermanno Candolfi, Guillaume Desoubeaux, Florent Morio, Christelle Pomares, Meja Rabodonirina, Alexandre Loywick, Sophie Merlin, Eric Viscogliosi, Magali Chabé

**Affiliations:** 1 GENES DIFFUSION, Douai, France; 2 PEGASE-Biosciences, Institut Pasteur de Lille, Lille, France; 3 Univ. Lille, CNRS, Inserm, CHU de Lille, Institut Pasteur de Lille, U1019 - UMR 8204 - CIIL - Center for Infection and Immunity of Lille, Lille, France; 4 Centre AZM pour la Recherche en Biotechnologies et ses applications, Université Libanaise, Laboratoire de Microbiologie Santé et Environnement, Tripoli, Lebanon.; 5 Direction de la Recherche Médicale, GHICL, Lille, France.; 6 Département de Parasitologie-Mycologie, CHU de Lille, Lille, France.; 7 Laboratoire de Parasitologie-Mycologie, CHU Gabriel-Montpied, Clermont-Ferrand, France.; 8 Clermont Université, Université Blaise Pascal- Université d’Auvergne-CNRS, UMR 6023, Laboratoire Microorganismes: Génome et Environnement, Clermont- Ferrand, France.; 9 Laboratoire de Parasitologie-Mycologie, CHU de Montpellier, CNRS UMR 5290/IRD 224/UM1, Université de Montpellier 1, Montpellier, France.; 10 Laboratoire de Microbiologie, CHU de Nîmes, Nîmes, France.; 11 Laboratoire de Parasitologie-Mycologie, CHU de Besançon, Besançon, France.; 12 Laboratoire de Mycologie-Parasitologie, AP-HP Hôpital Henri Mondor, Créteil, France.; 13 Institut de Parasitologie et de Pathologie Tropicale de Strasbourg, Université de Strasbourg, Hôpitaux Universitaires de Strasbourg, Strasbourg, France.; 14 Service de Parasitologie-Mycologie-Médecine Tropicale, CHU de Tours/CEPR Inserm U1100 Equipe 3, Université François- Rabelais de Tours, Tours, France.; 15 Laboratoire de Parasitologie-Mycologie, Institut de Biologie, CHU de Nantes, Département de Parasitologie et Mycologie Médicale, EA1155-IICiMed, Université de Nantes, Nantes, France.; 16 Laboratoire de Parasitologie-Mycologie CHU de Nice, C3M Inserm U1065, Université de Nice Sophia Antipolis, Nice, France.; 17 Service de Parasitologie, Hospices Civils de Lyon, Lyon, France.

## Abstract

Alterations in the composition of commensal bacterial populations, a phenomenon known as dysbiosis, are linked to multiple gastrointestinal disorders, such as inflammatory bowel disease and irritable bowel syndrome, or to infections by diverse enteric pathogens. *Blastocystis* is one of the most common single-celled eukaryotes detected in human faecal samples. However, the clinical significance of this widespread colonization remains unclear, and its pathogenic potential is controversial. To address the issue of *Blastocystis* pathogenicity, we investigated the impact of colonization by this protist on the composition of the human gut microbiota. For that purpose, we conducted a cross-sectional study including 48 *Blastocystis*-colonized patients and 48 *Blastocystis*-free subjects and performed an Ion Torrent 16S rDNA gene sequencing to decipher the *Blastocystis*-associated gut microbiota. Here, we report a higher bacterial diversity in faecal microbiota of *Blastocystis* colonized patients, a higher abundance of Clostridia as well as a lower abundance of Enterobacteriaceae. Our results contribute to suggesting that *Blastocystis* colonization is usually associated with a healthy gut microbiota, rather than with gut dysbiosis generally observed in metabolic or infectious inflammatory diseases of the lower gastrointestinal tract.

*Blastocystis* organisms are nonflagellated Stramenopiles found in a wide range of non-mammalian and mammalian hosts, including humans^1^,^2^. These protists are mainly transmitted by the faecal-oral route, and have been reported to be the most common single-celled eukaryotes detected in human faecal samples. Indeed, the prevalence of *Blastocystis* in humans varies from 0.5% to 30% in industrialized countries, and from 30% to 76% in developing countries^2^,^3^,^4^. Recently, a *Blastocystis* prevalence of 100% was found in a Senegalese population of children, which is the highest prevalence ever reported worldwide for this protist^5^.

With respect to its high prevalence in human populations, *Blastocystis* has largely been investigated under conditions in which it was believed to cause disease. However, the question of whether *Blastocystis* is a pathogen or a commensal of the human gut still has no definitive answer.

Prevalence data from different studies have implicated *Blastocystis* in various different intestinal and extraintestinal diseases, including inflammatory bowel disease (IBD), irritable bowel syndrome (IBS), and urticaria^1^,^2^,^6^,^7^,^8^,^9^. Moreover, its pathogenic potential in humans was recently questioned in *in vitro* studies together with *in silico* analysis of *Blastocystis* genomes that allowed the identification of potential virulence factors, such as cysteine proteases, and the proposal of a model for its pathogenesis^8^,^10^,^11^,^12^,^13^,^14^,^15^.

However, *Blastocystis* could also colonize the healthy human gut for long periods of time without resulting in symptomatic carrier status^16^,^17^. Some evidence even suggests that *Blastocystis* is a prevalent and diverse component of the microbiota in many individuals, as it has been detected with a high prevalence in healthy people^16^,^17^,^18^. Other studies have also demonstrated that *Blastocystis* was more prevalent in healthy controls compared with patients with ulcerative colitis or IBS^18^,^19^,^20^. Thus, the clinical significance of this common intestinal eukaryote remains unsettled and is likely to be correlated with various factors, including genetic variability and generation times of isolates and different host backgrounds as immune status.

As a complement to *in vitro*, *in vivo* and *in silico* studies, another way to address the issue of *Blastocystis* pathogenicity could be to investigate the effects of colonization by this protist on the composition of the gut microbiota. Indeed, it is now well established that alterations in the composition of commensal bacterial populations (a phenomenon known as ‘dysbiosis’) are linked to multiple intestinal diseases, such as IBD or IBS^21^,^22^, two potential *Blastocystis*-associated diseases. Furthermore, diverse enteric pathogens can induce significant perturbations in the microbiota or bloom during dysbiosis^23^. For example, intestinal dysbiosis was highlighted after acute *Toxoplasma gondii* gastrointestinal infection in mice^24^. The presence of gut microbiota is also essential for the pathogenic expression of certain enteric eukaryotes, such as *Entamoeba histolytica* or *Giardia duodenalis*^25^,^26^,^27^. Thus, the question arises of whether *Blastocystis* colonization is associated or not with a particular microbiota or with dysbiosis.

Since 2005, metagenomics supported by high-throughput sequencing (HTS) has unveiled a large part of the human microbiota. However, shotgun metagenomics still remains very expensive, and data analysis remains a challenging issue, due to both the size and complex structure of the data^28^. In parallel, targeted metagenomics, also called metagenetics^29^, focuses only on an informative genomic marker. For example, 16S rDNA metagenetics is commonly used to assess the bacterial microbiota. Thanks to HTS evolution and recent benchtop sequencer releases (Ion Torrent and MiSeq technologies), this culture-free application had become widespread in microbiology studies.

In this study, we investigated the gut microbial composition of *Blastocystis*-colonized patients. For this purpose, we performed a cross-sectional study, including 48 *Blastocystis*-colonized patients and 48 *Blastocystis*-free subjects, and used Ion Torrent 16S rDNA gene sequencing and bioinformatics analyses to profile and compare their gut bacterial communities. A higher bacterial diversity was found in *Blastocystis*-colonized patients compared to that identified in *Blastocystis*-free individuals. Moreover, *Blastocystis*-colonized patients exhibited a higher abundance of Clostridia at the class level, and a higher abundance of Ruminococcaceae and Prevotellaceae at the family level, whereas Enterobacteriaceae were enriched in *Blastocystis*-free patients. Based on these data, we suggest that *Blastocystis* colonization is not associated with the gut dysbiosis generally observed in metabolic or infectious intestinal diseases and commonly associated with inflammation of the lower gastrointestinal tract. Colonization by this protist could instead be associated with a healthy gut microbiota.

## Results

### Patient sampling

Our first goal was to select 48 samples positive for the protist and 48 *Blastocystis*-free samples among the available 788 faecal DNA samples, about 18% of which were positive for *Blastocystis*. In order to avoid selection bias in the choice of samples, the strategy described in the Materials and Methods section was followed.

After the odds ratio (OR), relative risks (RR) and logistic regression tests, six variables were found to be associated with the presence of *Blastocystis* in our cohort of patients as follows: hospital location, season of sampling, recent travels, presence of other parasites in faecal samples, fever, and abdominal pain. *Blastocystis* colonization was less frequent in winter than in summer (RR = 0.59 [0.45–0.81] and OR = 0.53 [0.36–0.77]) and higher in Lille than in other hospital locations (p-value < 0.01). *Blastocystis* was also more frequently present in faecal samples of patients having travelled recently (RR = 1.89 [1.36–2.62] and OR = 2.22 [1.46–3.37]) and presenting other faecal parasites (RR = 2.53 [1.81–3.54] and OR = 3.56 [2.11–5.99]). Presence of abdominal pain and fever were also found to be associated with *Blastocystis* colonization with RR scores of 1.52 [1.11–2.08] and 1.76 [1.10–2.81], respectively. These six variables were then used to compute the ranked *Blastocystis* colonization risk scores for each patient (see Supplementary Sheet S1). Therefore, the risk scores were used to select the 24 highest score values and the 24 lowest scores in each population of *Blastocystis*-positive or *Blastocystis*-free patients, thus determining 4 groups of 24 patients. Groups 1 and 2 were composed of *Blastocystis*-colonized patients and groups 3 and 4 included *Blastocystis*-free patients; groups 1 and 3 were linked in terms of low environmental and clinical risk factors, and groups 2 and 4 shared high risk factors (see Supplementary Sheet S2).

### Ion Torrent Sequencing and preprocessing of reads

The sequencing run of the 96 samples indexed on an Ion 318™ Chip resulted in approximately 1.1 GB of data with a mode reads of 403 bases. A total of 3,962,103 output sequence reads with an average of 42,603 reads per index and an average length of 272 bases per read were obtained (see Supplementary Table S1).

After the preprocessing workflow consisting of a high-quality filtering approach, the final read number was reduced by 31% (2,742,108 reads) and the median read number per index was 29,419, thus highlighting that this approach yielded high-quality sequence data from 16S rRNA gene amplicons (see Supplementary Table S1).

Independent-sample t-tests were conducted to compare the averages of read length, read number per index from output sequence data, and preprocessed read number per index, in both *Blastocystis*-colonized and *Blastocystis*-free patient samples. There were no significant differences between the two main groups for any variable, indicating that there was no technical bias in sequencing for the two main groups of patients (t-values = 0.49; 1.13; 1.77 and p = 0.62; 0.26; 0.08 for the three variables, respectively).

### *Blastocystis* colonization is associated with increased 16S rRNA gene sequence diversity of gut microbiota

**Figure 1:**
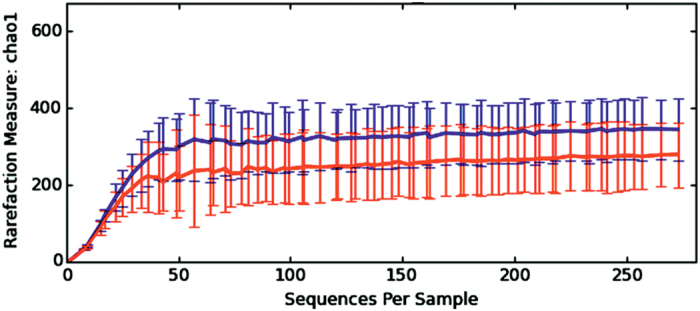
Rarefaction curve calculated for Chao1 index demonstrating the higher bacterial diversity found among *Blastocystis*-colonized patients. The blue line indicates *Blastocystis*-colonized patients and the red line indicates *Blastocystis*-free individuals.

Alpha-diversity rarefaction curves obtained by plotting the Chao1, Observed OTUs, Shannon and Simpson diversity indexes against the number of sequences per sample (Fig. 1) showed that the sequencing depth encompassed the full extent of phylotype richness in each of the samples, and a large part of the diversity of the samples was detected.

**Figure 2:**
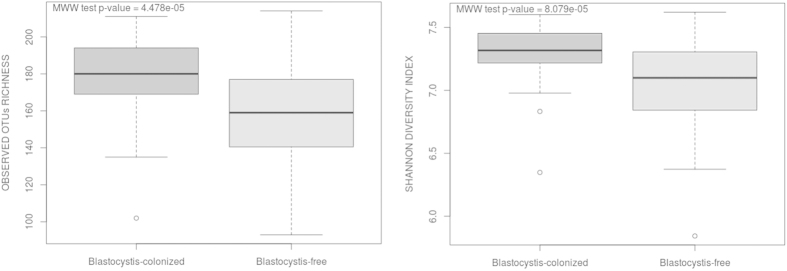
Boxplots of observed OTUs richness and Shannon diversity indexes distinguishing between patients colonized or not by *Blastocystis*. Statistical analyses were performed using the Mann-Whitney-Wilcoxon (MWW) test. Plotted are interquartile ranges (IQRs; boxes), medians (dark lines in the boxes), and the lowest and highest values within 1.5 times IQR from the first and third quartiles (whiskers above and below the boxes). Both alpha-diversity metrics were calculated using 273 normalized sequences per sample.

A Mann-Whitney-Wilcoxon test was performed to compare the estimated richness and diversity indexes between the *Blastocystis*-colonized and not colonized patient samples. A higher bacterial diversity in faecal microbiota of patients colonized with *Blastocystis* was detected. Indeed, Shannon and Simpson diversity indexes demonstrated that the faecal microbiota diversity in colonized patients was significantly higher than that observed in *Blastocystis*-free subjects (p < 0.01) (Fig. 2, Table 1). Likewise, observed OTUs and Chao1 richness indexes in colonized patients were significantly higher than those in *Blastocystis*-free subjects (p < 0.01) (Figs 1 and 2, Table 1). These four indexes were strongly correlated (data not shown). Collectively, these data point towards a greater microbial diversity in the faecal microbiota of *Blastocystis*-colonized patients.

To explore the differences in the overall microbial community composition across the two main groups of patients, the phylogenetic taxonomic Bray–Curtis dissimilarities were calculated.

**Figure 3:**
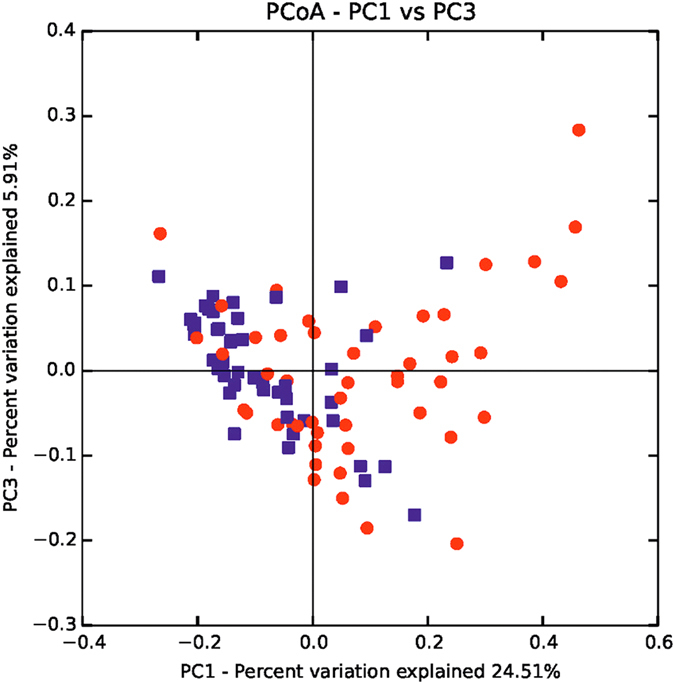
PCoA of the microbial communities in *Blastocystis*-colonized and *Blastocystis*-free patient samples. The blue dots indicate *Blastocystis*-colonized patients and the red dots indicate *Blastocystis*-negative individuals.

When the subjects were classified based on their *Blastocystis* colonization status, the PCoA plots obtained from the beta diversity calculation in QIIME demonstrated a relative clustering of samples, whereby the scores for PC1 and PC3 account for 30% of the variance in the data (Fig. 3). This difference between the bacterial communities was significant, as determined using the ANOSIM nonparametric statistical test analysis of similarity, where R = 0.112 (p = 0.001).

### Bacterial microbiota composition of *Blastocystis*-positive faecal samples

The composition of bacterial faecal microbiota of *Blastocystis*-colonized and *Blastocystis*-free patients was further examined. Initially, the OTU relative abundances from bacterial taxa in each patient’s faecal sample were plotted (see Supplementary Fig. S1). At the phylum level, Firmicutes, Bacteroidetes and Proteobacteria were the most predominant phyla in all patients’ faecal samples (see Supplementary Fig. S1).

The differences in the relative abundances of OTUs in faecal microbiota of colonized and not colonized patients were further explored using the Mann-Whitney-Wilcoxon test at different taxa levels. The taxonomic differences between the two groups were also compared using read counts normalized by DESeq2 (White’s nonparametric t-test corrected by Benjamini-Hochberg False Discovery Rate multiple test) in STAMP v 2.0.9^30^.

At the class level, three bacterial taxa were present at different levels in colonized patients compared to *Blastocystis*-free subjects (p < 0.05 for both Mann-Whitney-Wilcoxon (MWW) test and White’s nonparametric t-test (W)). Clostridia had a significantly higher relative abundance in colonized patients’ samples compared with the *Blastocystis*-free groups (MWW, p = 0.0133; W, p = 0.049), while relative abundance of Bacilli was significantly lower in colonized patients than in the *Blastocystis*-free subjects (MWW, p = 0.0051; W, p = 0.00612) (see Supplementary Fig. S1). Mollicutes were also more abundant in colonized patients (p < 0.05 in both tests).

**Figure 4:**
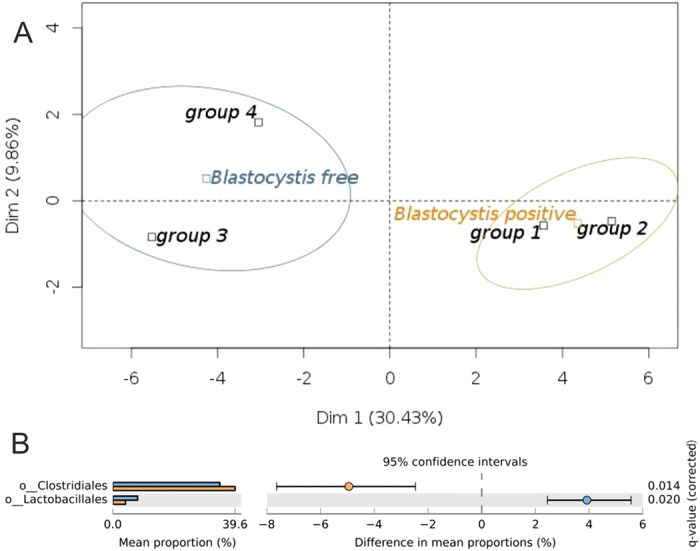
Analyses of the bacterial microbiota composition at the order-level taxonomic rank. (**A**) PCA plot comparing the four patient groups according to their microbiota patterns for the 5 most abundant microbial communities at the order-level taxonomic rank. Groups 3 and 4 define the *Blastocystis*-free cluster (in blue); groups 1 and 2 define the *Blastocystis*-positive cluster (in orange). (**B**) Proportion of sequences assigned to each main group at the order-level taxonomic rank (level 4) for Clostridiales and Lactobacillales illustrated using STAMP, along with means for each group and the significance of the difference in mean proportions using White’s nonparametric t-test with Benjamini-Hochberg FDR multiple test correction. The blue and orange bars represent *Blastocystis*-free patients and *Blastocystis*-positive patients, respectively.

At the order taxonomic level, a PCA plot (Fig. 4(a)) was produced using the 5 most abundant taxa (normalized counts) (i.e. Clostridiales, Lactobacillales, Bacteroidales, Erysipelotrichales and Burkholderiales). More than 40% of the total variance was explained using the first two principal components. This graphic shows that, on one hand, groups 3 and 4, the two groups constituting the *Blastocystis*-free cluster, had a homogeneous microbiota pattern that is relatively distinct from, on the other hand, groups 1 and 2 that constitute a homogeneous *Blastocystis*-positive cluster. The relative abundance of the 5 predominant bacteria orders for these 4 groups of patients was quite similar, ranging from 68.58% to 71.12%, respectively, for groups 4 and 3. Among these 5 orders, only two, Lactobacillales and Clostridiales, could be considered to be statistically differentially abundant between *Blastocystis*-free and *Blastocystis*-positive main groups (Fig. 4(b)). Clostridiales, which represented the major abundant order, were significantly more abundant in colonized patients (MWW, p = 0.0133; W, p = 0.014), and Lactobacillales were more profuse in *Blastocystis*-free individuals (MWW, p = 0.001; W, p = 0.020) (see Supplementary Fig. S1, Fig. 4(b)). These results were confirmed by performing a Mann-Whitney-Wilcoxon test analysis on OTU relative abundances in the faecal microbiota of group 1 vs. 3 or group 2 vs. 4 patients (p < 0.05) (data not shown).

**Figure 5:**
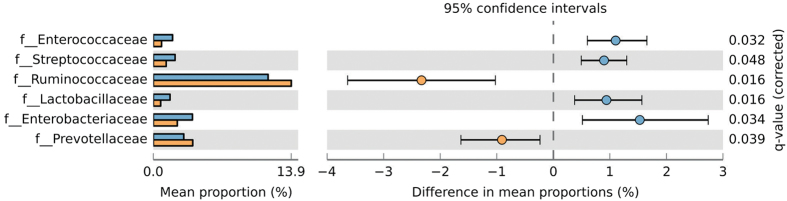
Proportion of sequences assigned to each main group at the family taxonomic rank (level 5), along with the means for each group and significance of difference in mean proportions using White’s nonparametric t-test with Benjamini-Hochberg FDR multiple test correction, illustrated using STAMP. Significant differences (q-value < 0.05) are represented here between the two main groups. Blue and orange bars represent *Blastocystis*-free patients and *Blastocystis*-colonized patients, respectively.

At the family level, the bacterial composition of the samples also showed differences between the two main groups. Ruminococcaceae and Prevotellaceae families were prevalent in colonized patients (p < 0.05 in both tests), while Enterococcaceae, Streptococcaceae, Lactobacillaceae and Enterobacteriaceae were enriched in *Blastocystis*-free subjects (p < 0.05) (see Supplementary Fig. S1, Fig. 5).

**Figure 6:**
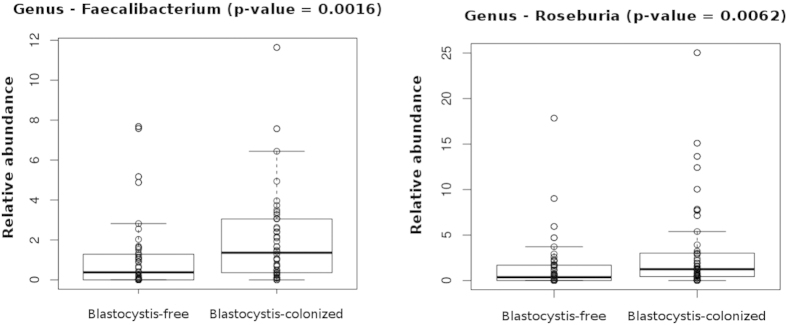
Relative abundances of OTUs of *Faecalibacterium* and *Roseburia* genera that differ significantly between *Blastocystis*-colonized and *Blastocystis*-free patients. The Mann-Whitney-Wilcoxon test was used to evaluate the two groups.

Differences in relative abundances of OTUs in faecal microbiota of *Blastocystis*-positive or *Blastocystis*-negative patients have also been identified at the genus level. The OTU relative abundances of several bacterial taxa of Clostridiales like the genera *Acetanaerobacterium*, *Acetivibrio*, *Coprococcus*, *Hespellia*, *Oscillibacter*, *Papillibacter*, *Sporobacter* and *Ruminococcus* were higher in patients colonized with *Blastocystis* than in *Blastocystis*-free individuals (p < 0.05). The OTU relative abundance of *Prevotella* was also higher in colonized patients (p < 0.05). *Roseburia* and *Faecalibacterium*, two well-known butyrate-producing bacterial genera that seem to play a key role in gut homeostasis^31^, had a significantly higher abundance in colonized than in *Blastocystis*-negative patients (Fig. 6).

## Discussion

Despite numerous studies reporting that *Blastocystis* can cause digestive symptoms, the clinical significance and pathogenic potential of this widespread protist remain unclear. Determining whether or not *Blastocystis* colonization is associated with gut dysbiosis could be therefore important for understanding these scientific issues.

In 2014, Nourrisson *et al.* investigated the potential impact of *Blastocystis* carriage on the enteric microbial community through qPCR quantification of eight major bacterial groups in patients with IBS, or control subjects without any gastrointestinal disorders^32^. *Blastocystis* carriage was shown to be associated with a decrease in *Bifidobacterium* in male IBS patients with constipation. In the control group, no significant differences between both *Blastocystis*-positive and *Blastocystis*-negative patients were observed^32^.

More recently, Andersen *et al.* performed a retrospective analysis of metagenomic data obtained by the MetaHIT Consortium to detect microbial eukaryotic DNA signatures, by applying a co-abundance binning method for constructing CAGs (co-abundance gene groups)^33^. In this study, the presence of *Blastocystis* was negatively correlated with the *Bacteroides*-driven enterotype of the gut microbiome^33^.

Despite these previous surveys, with results that are self-limiting due to the methodology used, no study was conducted, as far as we are aware, to accurately analyse the *Blastocystis*-associated microbiota. For the first time, the *Blastocystis*-associated faecal bacterial microbiota was successfully deciphered using a 16S rDNA gene-based approach and by comparing the microbiota of *Blastocystis*-colonized patients and *Blastocystis*-free individuals.

From a technical point of view, assessing the distribution of 16S rRNA gene sequences within a biological sample, called “metagenetics”^29^, represents the current state of the art for the determination of human gut microbiota composition. Our study confirms the accuracy and efficiency of the 16S rDNA gene sequencing approach using the Ion Torrent PGM sequencing platform to determine the composition of faecal microbiota. Recently, this HTS technology has already been successfully used to investigate the gut microbiota of patients with systemic lupus erythematosus^34^ or healthy subjects^35^. In our study, one sequencing run performed with 96 DNA samples indexed in one Ion 318™ Chip was efficient in obtaining enough high-quality sequence data (i.e. a median read number per index of 29,419) in order to compare the microbiota profiles of 46 *Blastocystis*-colonized patients and 47 *Blastocystis*-free subjects.

Although efforts to define what “normal” gut microbiota means are still progressing, it is well known that the level of diversity and bacterial species composition of the microbiota of healthy individuals differ from those of patients suffering from many metabolic or infectious diseases. The dysbiosis of the intestinal microbiota related to metabolic or infectious diseases like IBS, IBD or enteric pathogens infections^21^,^22^,^23^, that is commonly associated with inflammation of the lower GI tract, is typified by a reduction in bacterial diversity, a decreased abundance of Clostridia and a bloom of facultative anaerobic Gammaproteobacteria like Enterobacteriaceae^36^,^37^.

In our study, an unexpectedly higher bacterial diversity was found in *Blastocystis*-colonized patients compared to that identified in *Blastocystis*-free individuals. Moreover, at the class level, *Blastocystis*-colonized patients exhibited a higher abundance of Clostridia. In colonized patients, Ruminococcaceae and Prevotellaceae families were also more abundant than in *Blastocystis*-negative patients, whereas Enterobacteriaceae were enriched in these *Blastocystis*-free patients. Additionally, the genera *Faecalibacterium* and *Roseburia*, known to contain butyrate-producing bacteria, had a significantly higher abundance in *Blastocystis*-positive patients. Interestingly, butyrate is considered one of the most important metabolites for maintaining colonic health in humans, as it serves as the major energy source of colonic epithelial cells, has anti-inflammatory properties, and regulates gene expression, differentiation and apoptosis in host cells^38^,^39^,^40^. Significant reductions in the abundance of these bacteria have been identified as markers of dysbiosis in patients with ulcerative colitis or Crohn’s disease^37^,^41^,^42^.

Altogether, our results seem to go against the idea that *Blastocystis* is associated with gut dysbiosis related to metabolic or infectious inflammatory diseases of the gastrointestinal tract, and tend to hypothesize that *Blastocystis* colonization is rather associated with a healthy gut microbiota, as suggested previously^17^,^43^.

To our knowledge, the only parasites that have been associated with an increased bacterial diversity are helminths. Broadhurst *et al.* showed that exposure of rhesus monkeys affected by idiopathic chronic diarrhea to the whipworm *Trichuris trichiura* led to clinical improvement in macaques, and that helminth treatment promoted the restoration of the diversity of mucosal microbial communities^44^. In humans, in a more recent study comparing the composition and diversity of bacterial communities from the faecal microbiota of Malaysian individuals colonized or not by helminths, helminth colonization was associated with greater bacterial species richness^45^. Interestingly, the ability of gastrointestinal nematodes to modulate intestinal inflammation and improve the pathology of chronic intestinal diseases has even led to the development of helminthic therapy. In a recent paper, Cantacessi *et al.* suggested that hookworms administered to patients with chronic inflammatory diseases of the intestinal tract could exert their therapeutic effect, at least in part, by maintaining microbial diversity and thereby restoring microbial homeostasis in the gut^46^.

To date, *Blastocystis* could thus be the only known protist whose colonization would be associated with increased faecal bacteria diversity, and could perhaps even be a beneficial component for gut homeostasis. Interestingly, Lukeš *et al.* have already considered the use of some protists like *Blastocystis* for their potential to stimulate an immune response in a manner beneficial for humans with allergies or IBD^47^, like it was described for some intestinal helminths. If these studies are confirmed, the interaction between *Blastocystis* and humans will need to be recast as commensalistic or even mutualistic, at least under certain conditions.

Although the concept of enterotypes has been debated^48^,^49^, analysis of metagenomic data obtained by the MetaHIT Consortium has revealed differences in *Blastocystis* prevalence among the enterotypes used for gut microbiome stratification^33^. *Blastocystis* seems to be common in patients with *Prevotella*- and *Ruminococcus*-driven enterotypes, whereas individuals with gut microbiota skewed towards *Bacteroides* are less prone to be carriers of *Blastocystis*^33^. The aim of our study was not to determine the enterotypes of patients, but it seems interesting to point out that the OTU relative abundance of *Ruminococcus* and *Prevotella* were higher in *Blastocystis*-positive patients than in *Blastocystis*-negative individuals.

This first metagenetic study designed to decipher *Blastocystis*-associated microbiota was deliberately not focused on patients with IBD or IBS pathologies, for which *Blastocystis* could be implicated in the physiopathology^1^,^2^,^7^,^8^,^9^. Indeed, of the 96 patients included in our study, none have Crohn’s disease or ulcerative colitis, and only six were IBS patients (2 patients in groups 1 and 3, and 1 patient in groups 2 and 4). However, this study proves that our methodology, based on 16S rDNA gene sequencing in the Ion Torrent PGM platform and bioinformatics analysis of the data, provides a rapid, simple, cost-effective and accurate system with which to compare, in the future, the microbiota of patients with IBD or IBS, colonized or not with *Blastocystis*.

Finally, our work highlighted for the first time the greater bacterial diversity of faecal samples of *Blastocystis*-colonized patients and pointed out that *Blastocystis* colonization seems not to be associated with the gut dysbiosis usually observed in metabolic or infectious diseases of the gastrointestinal tract. Nevertheless, this type of isolated study cannot determine if there are causal relationships between the parasite and gut microbiota. This is like the chicken and the egg question: which came first? Is this finding due to a reduced ability of *Blastocystis* to establish itself in the microbiota of patients with gut dysbiosis, or is *Blastocystis* playing a role in structuring microbiota communities and actively contributing to intestinal homeostasis? To address this, further longitudinal metagenetic studies in humans are required. Our results also highlight the urgent need for developing an effective and reproducible *Blastocystis* animal model. Comparison of the microbiota of *Blastocystis*-free animals before and during the colonization by the protist could continue to advance the frontiers of knowledge in the understanding of the functional impact of *Blastocystis* on the bacterial microbiota.

## Materials and Methods

### Ethics statement

This study was approved by the Research Ethics Committee “Comité de Protection des Personnes Sud-Est 6, France” (reference number 2015/CE82), which waived the requirement for informed consent because the experiments did not result in additional constraints for the patients. All the methods used in the study were carried out in accordance with the approved guidelines (World Medical Association’s (WMA) Declaration of Helsinki-Ethical Principles for Medical Research Involving Human Subjects).

### Study subjects

The initial epidemiological survey enrolled 788 patients followed up for different pathologies (with/without gastrointestinal symptoms), at 11 French hospitals (Lille, Clermont-Ferrand, Strasbourg, Nimes, Besançon, Lyon, Tours, Nantes, Montpellier, Nice and Créteil) (D. El Safadi, A. Cian, C. Nourrisson, B. Pereira, C. Morelle, P. Bastien, A. P. Bellanger, F. Botterel, E. Candolfi, G. Desoubeaux, L. Lachaud, F. Morio, C. Pomares, M. Rabodonirina, I. Wawrzyniak, F. Delbac, N. Gantois, G. Certad, L. Delhaes, P. Poirier, and E. Viscogliosi, submitted for publication). In this previous study, one stool sample was obtained from each patient, either in summer or winter, and tested after DNA extraction with the QIAamp DNA stool Mini Kit (Qiagen) for the presence of *Blastocystis* using a real-time PCR assay^50^. The overall prevalence of *Blastocystis* was shown to be 18.1% (143/788). A standardized questionnaire was designed to collect information about each participating subject (sex, age, profession, recent travels and destinations, exposure to pets) as well as clinical data, especially regarding the presence of digestive symptoms (diarrhea, vomiting, bloating, constipation and abdominal pain), and diagnosis of IBS or IBD. In addition, the observation of intestinal protozoans, fungi, and helminths by direct-light microscopy of faecal smears was also recorded, as well as digestive diseases of bacterial origin.

The first step of the present study was thus to select 48 samples positive for the protist and 48 *Blastocystis*-free samples among the available 788 faecal DNAs. In order to reduce the likelihood of selection bias in the choice of patients, the following strategy was used. The odds ratio (OR) and relative risks (RR) were calculated to quantify how strongly different Boolean variables (including the sampling season and all variables cited above) were associated with the presence or absence of *Blastocystis* in the population of 788 individuals. A logistic regression test was also performed for non-Boolean variables like hospital locations. A forward stepwise logistic regression method using AIC (Akaike information criterion) criterion was then used to select the variables and build the optimal model in order to compute a *Blastocystis* colonization risk score (see Supplementary Sheet S1). The risk scores of each patient were then ranked, a high score being indicative of high *Blastocystis* colonization susceptibility.

Of the 143 *Blastocystis*-positive patients, those with the 24 highest risk scores and those with the 24 lowest scores were selected. The same selection was performed among the 645 *Blastocystis*-free individuals. Therefore, 96 DNA samples corresponding to two groups of 48 patients, positive or negative for *Blastocystis*, comparable in terms of *Blastocystis* colonization risk score, and indirectly in terms of clinical and environmental variables, were used in our study. All statistical analyses were performed in the R environment for statistical computing^51^.

### DNA extraction monitoring

Faecal DNAs corresponding to the 96 patients were randomly distributed in a 96-well microplate and the total DNA concentration of each sample was measured using the Quant-iT PicoGreen dsDNA assay (Invitrogen). The global 16S gene DNA copy number was measured by a SybrGreen quantitative PCR method adapted from Maeda *et al.*^52^, which allows both inhibition effect estimation and DNA concentration adjustment. The reaction mixture (15 μL) for the SybrGreen assay performed in RotorGene (Corbett Life Science) contained 2X Brilliant III SybrGreen qPCR Mastermix (Stratagene), primers (GTGSTGCAYGGYTGTCGTCA, Univ16S_1048-1067 as the forward primer and ACGTCRTCCMCACCTTCCTC, Univ16S_1175_1194 as the reverse primer) with a final concentration of 560 nM and 2 μL of DNA extract as the template. The amplification conditions were 3 min at 94 °C, 45 cycles of 15 s at 94 °C for denaturation, 22 s at 60 °C for annealing and extension, followed by a melting curve from 54 °C to 95 °C.

### Metagenetic high-throughput sequencing

The sequence regions of the 16S rRNA gene spanning variable regions V3–V5 were then amplified using the broad-range forward primer For16S_519, CAGCMGCCGCGGTAATAC and the reverse primer Rev16S_926, CCGTCAATTCMTTTGAGTTT. Library preparations for amplicons sequencing were performed in a final volume of 100 μL containing 1X PCR buffer, 2 mM MgSO4, 1 U of DNA High Fidelity Taq Polymerase (Invitrogen), 625 nM of each barcoded primer (IDT), 250 μM of each dNTP (Invitrogen), and a concentration-adjusted DNA sample. Each sample was taken following two PCRs, one with the sequencing adapter linked to the forward primer, the other with the sequencing adapter linked to the reverse primer. The two resulting PCR products were equimolarly pooled after DNA purification with NucleoFast^®^ 96 PCR (Macherey Nagel), followed by a Quant-iT PicoGreen ds DNA quantification. The 96-barcoded bidirectional library was sequenced through PGM, Ion Torrent (Life Technologies) with the Ion 318™ Chip and Ion PGM™ 400 Sequencing Kit (Life Technologies), following the recommended protocol.

### Sequence-based microbiota analysis

To analyse the Ion Torrent sequencing data, a homemade pipeline was developed using various publicly available tools such as Mothur^53^ or EspritTree^54^, databases such as the Silva small subunit RNA database and Ribosomal Database Project (RDP) and Perl/Python scripts (find more information on http://www.pegase-biosciences.com/pub_2014/#ECCB). The whole pipeline was integrated into a Galaxy server^55^ and could be divided into two main modules.

#### Preprocessing of FastQ files

The first module corresponded to a preprocessing step producing a curated and filtered collection of reads using Mothur tools^53^. Firstly, all reads shorter than 150 bases were removed. The remaining sequences were then trimmed to remove the erroneous homopolymers generated by the Ion Torrent PGM sequencer, with a maximum limit for homopolymer length set to 20. Once this filtering was applied, duplicated sequences were grouped so as to save computing time during the alignment and clustering steps. The sequences were then aligned against the Silva database (release 102)^56^ provided with Mothur^53^. Sequences with alignments under 100 bases were filtered out.

#### Clustering analysis and OTU classification

In the second module, Esprit-Tree software was used for OTU clustering^54^. Briefly, a partition tree was computed using the PbpCluster tool from the Esprit-Tree suite and a consensus sequence was generated for each OTU cluster using a distance level of 0.05. Then, the classification of the reference sequences of each OTU was determined using Mothur against the RDP taxonomy in the Silva database^57^. The output results were grouped into a standard BIOM-formatted table and used for the subsequent analyses^58^.

#### Read count normalization

The total read counts were normalized using the DESeq2 package^59^ integrated into QIIME^60^ before conducting downstream analyses, following previous recommendations to avoid rarefaction of the read count data^61^.

#### Secondary analysis

Alpha (within a community) and beta (between communities) diversity metrics, as well as taxonomic community assessments, were produced using QIIME 1.8 scripts on the normalized read counts. The following indexes were calculated for each sample: Chao1 bias-corrected, observed OTUs, Shannon and Simpson. Alpha-diversity rarefaction curves were produced by plotting these several diversity metrics against the number of sequences considered from a sample. Alpha diversity indexes of *Blastocystis*-colonized patients were compared to *Blastocystis*-free subjects with the Mann-Whitney-Wilcoxon using R software^51^, at a depth of 273 sequences per sample. To explore the differences in overall microbial community composition across the two groups of patients, the taxonomic Bray-Curtis dissimilarities were calculated after additional filtering of the normalized read counts to remove singletons. The QIIME beta_diversity_through_plots Python script also produced a Principal Coordinates Analysis (PCoA) plot in which the Bray-Curtis dissimilarities between the samples were used to visualize the differences among groups of samples. Environments producing distinct clustering of samples were subjected to significance testing using the nonparametric statistical analysis of similarity (ANOSIM) implemented in the vegan R package^51^ with 999 permutations.

The microbiota features obtained from normalized read counts of *Blastocystis*-colonized patients were compared to those of *Blastocystis*-negative subjects with White’s nonparametric t-test (Benjamini-Hochberg with False Discovery Rate multiple test correction) using STAMP v 2.0.9^30^. All tests for significance were two-sided, and corrected p-values (i.e. q-values) <0.05 were considered statistically significant. In parallel, the differences in the relative abundances of the OTUs in faecal microbiota of colonized or not colonized patients were explored using the Mann-Whitney-Wilcoxon test in the R statistics package^51^ at different taxa levels.

To visualize the overall bacterial microbiota composition among the different groups, a Principal Component Analysis (PCA) plot was produced using the 5 most abundant taxa at the order taxonomic rank and the R FactoMineR package^62^.

## Additional Information

**How to cite this article**: Audebert, C. *et al.* Colonization with the enteric protozoa *Blastocystis* is associated with increased diversity of human gut bacterial microbiota. *Sci. Rep.* **6**, 25255; doi: 10.1038/srep25255 (2016).

## Supplementary Material

Supplementary Information

Supplementary dataset 1

## Figures and Tables

**Table 1:** Means of bacterial richness and diversity estimates in groups of patients colonized or not with *Blastocystis*.

Group of patients	Richness estimator				Diversity index			
	ObservedOTUs	p-value^a^	Chao 1	p-value^a^	Shannon	p-value^a^	Simpson	p-value^a^
*Blastocystis*-colonized	178.85	4.48e-05	340.20	1.66e-04	7.30	8.08e-05	0.78	0.0033
*Blastocystis*-free	157.64		273.22		7.05		0.73	

Statistical analysis to compare both main groups was performed using the Mann–Whitney-Wilcoxon test. *Significance testing of richness and diversity estimators was conducted using Mann–Whitney-Wilcoxon test as implemented in the stats package in R^50^.
