# Supplementary material for: Colonization with the enteric protozoa Blastocystis is associated with increased diversity of human gut bacterial microbiota
Source: Sci Rep. 2016 May 5;6:25255. doi: 10.1038/srep25255 (PMC4857090; doi:10.1038/srep25255)
Supplement: Supplementary Information [file srep25255-s1.pdf]

# Colonization with the enteric protozoa *Blastocystis* is associated with increased diversity of human gut bacterial microbiota

Christophe Audebert[1,2,+], Gaël Even[1,2,+], Amandine Cian[3], The *Blastocystis* Investigation Group[‡], Alexandre Loywick[1,2], Sophie Merlin[1,2], Eric Viscogliosi[3], and Magali Chabé[3,*]

[1]GENES DIFFUSION, Douai, France

[2]PEGASE-Biosciences, Institut Pasteur de Lille, Lille, France

[3]Univ. Lille, CNRS, Inserm, CHU de Lille, Institut Pasteur de Lille, U1019 - UMR 8204 - CIIL - Center for Infection and Immunity of Lille, Lille, France

*corresponding author: magali.chabe@univ-lille2.fr

[+]These authors contributed equally to this work.

[‡]See the list of The *Blastocystis* Investigation Group members at the end of the manuscript.

**Supplementary information:**

**Supplementary Figure S1**: Faecal bacterial communities present in the different samples (DNA samples from patients' groups 1, 2, 3 and 4) analyzed in this study. Plots of relative abundances of OTUs at four taxonomic levels: phylum (a), class (b), order (c) and family (d). The most abundant and interesting taxa are labeled with arrows.

**Supplementary Table S1**: Reads metrics from output sequence data (PGM quality-approved, trimmed and filtered data) and mean number of preprocessed reads per index after the high-quality filtering approach. * Three outlier samples (indexes 18, 63 and 50 belonging to groups 1, 2 and 3 respectively), were discarded before the subsequent bioinformatics analyses.

**Supplementary Figure S1**. Faecal bacterial communities present in the different samples (DNA samples from patients' groups 1, 2, 3 and 4) analyzed in this study. Plots of relative abundances of OTUs at four taxonomic levels: phylum (a), class (b), order (c) and family (d). The most abundant and interesting taxa are labeled with arrows.

a)

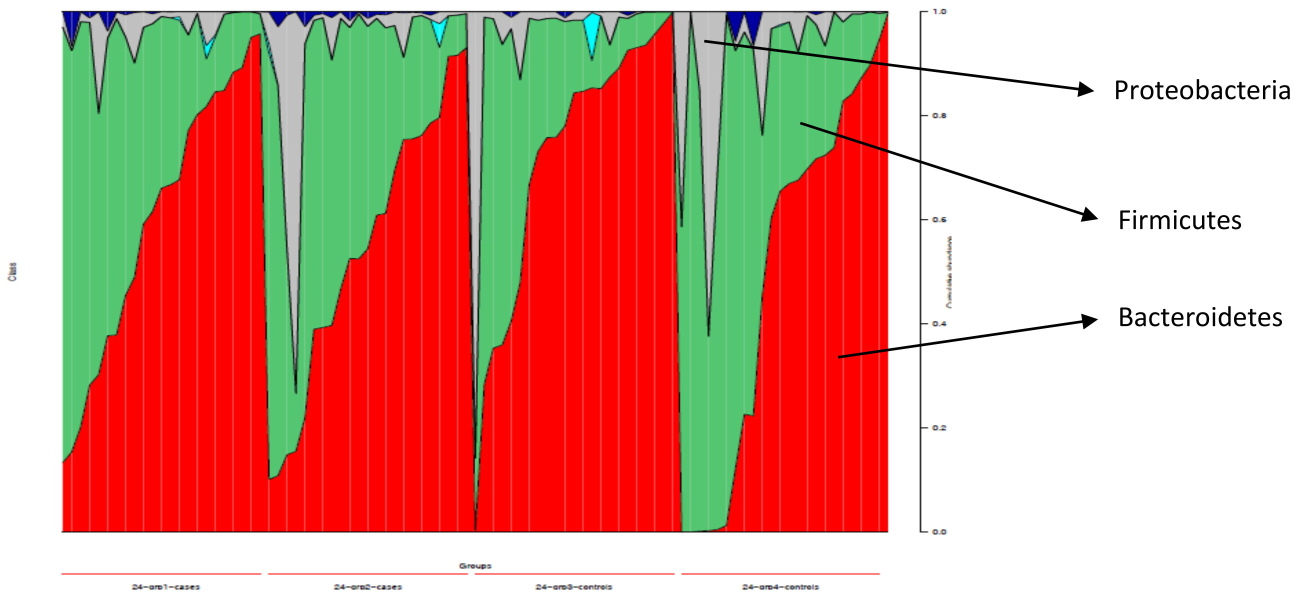


b)

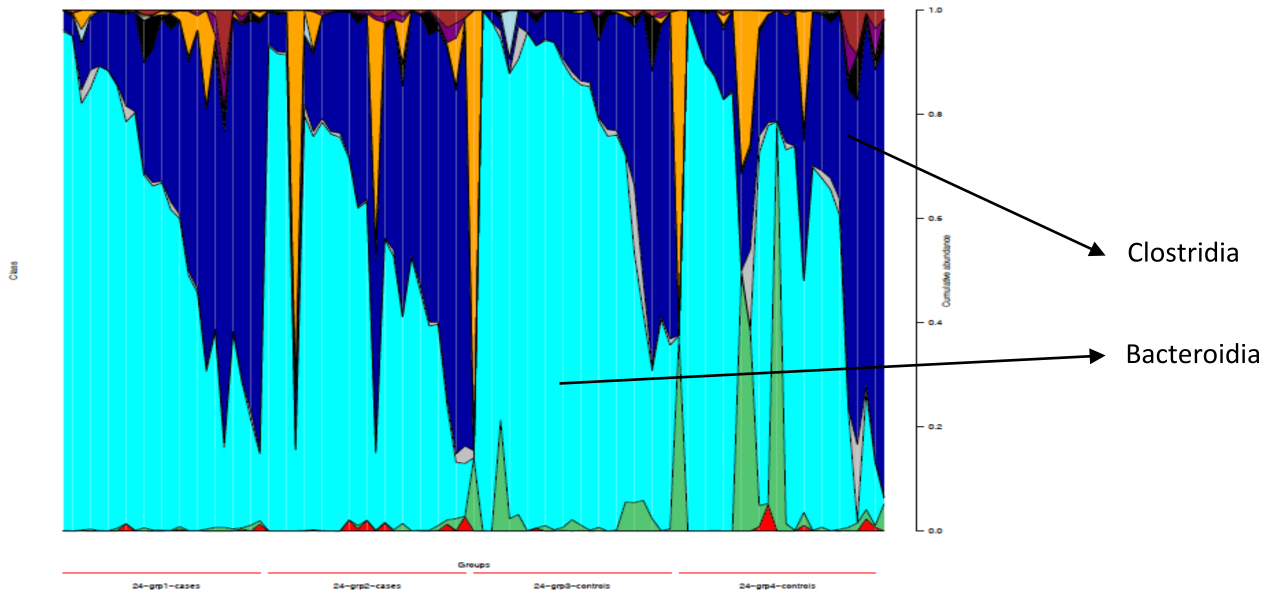

c)

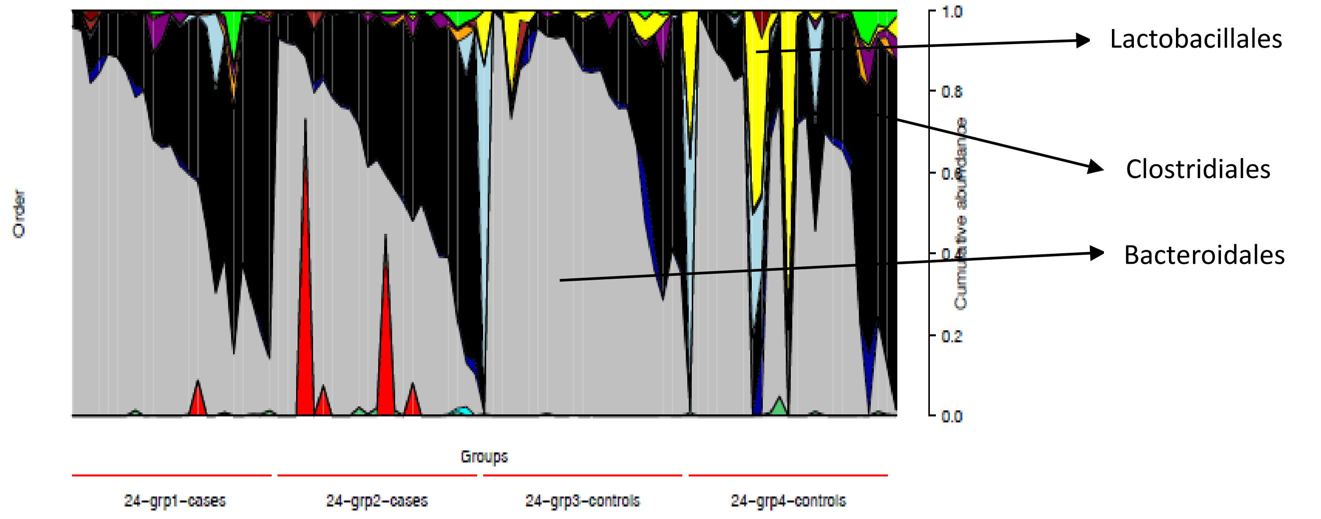

Order
Cumulative abundance
1.0
0.8
0.6
0.4
0.2
0.0
Lactobacillales
Clostridiales
Bacteroidales
Groups
24-grp1-cases
24-grp2-cases
24-grp3-controls
24-grp4-controls


d)

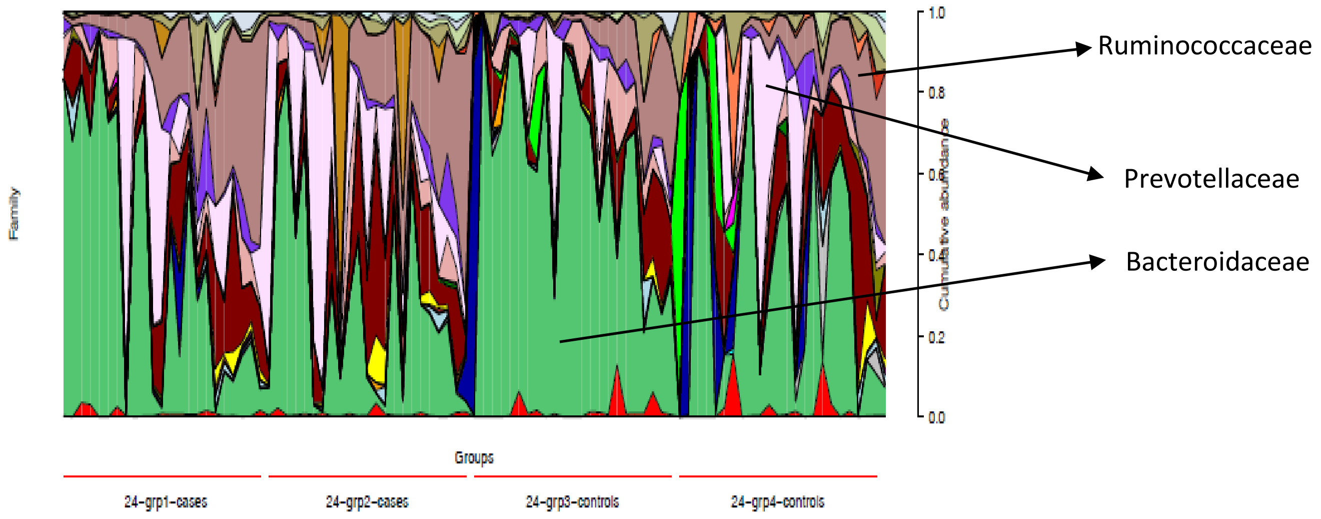

Family
Cumulative abundance
1.0
0.8
0.6
0.4
0.2
0.0
Ruminococcaceae
Prevotellaceae
Bacteroidaceae
Groups
24-grp1-cases
24-grp2-cases
24-grp3-controls
24-grp4-controls

**Supplementary Table S1.** Reads metrics from output sequence data (PGM quality-approved, trimmed and filtered data) and mean number of preprocessed reads per index after the high-quality filtering approach. * Three outlier samples (indexes 18, 63 and 50 belonging to groups 1, 2 and 3 respectively), were discarded before the subsequent bioinformatics analyses.

| | Mean read length (bases) | Median read length (bases) | Minimum read number/index | Mean read number/ index | Median read number / index | Mean preprocessed reads number/index |
|---|---|---|---|---|---|---|
| All reads[a] | 272.67 | 273 | 14,617 | 42,603.26 | 41,956.00 | 29,485.03 |
| Reads from *Blastocystis* -colonized patients | 271.98 | 274 | 14,617 | 43,604.02 | 44,533.50 | 30,541.19 |
| Reads from *Blastocystis*-free individuals | 273.34 | 272 | 21 039 | 41,623.79 | 41,057.00 | 28,451.34 |
